# Lessons learned during the sliding gantry CT implementation in a trauma suite

**DOI:** 10.1007/s00068-022-02080-0

**Published:** 2022-08-21

**Authors:** Benjamin Lucas, Matthias Meng, Wiebke Schirrmeister, Gerald Pliske, Felix Walcher, Jan Philipp Schüttrumpf

**Affiliations:** Department of Trauma Surgery, Otto-Von-Guericke University Magdeburg, Leipziger Str. 44, 39120 Magdeburg, Germany

**Keywords:** Emergency medicine, Trauma registry, Trauma suite, Whole-body computed tomography

## Abstract

**Purpose:**

Early detection of bleeding is important for managing trauma cases in the emergency department (ED). Several trauma suites are equipped with computed tomography (CT) scanners to reduce the time to CT. In the last decade, sliding gantry CT has been implemented in trauma suites, highlighting conventional techniques' advantages. We investigated the change in the time to CT and the challenges faced during the implementation.

**Methods:**

Trauma suite treatments with a conventional CT scanner between January and December 2016 formed the control group. From January to April 2017, trauma suites were modified, and treatment was outsourced to an interim trauma suite. By May 2017, trauma suites were equipped with a sliding gantry CT scanner. Treatments from May to July 2017 formed the transition group, and those from August to December 2017 formed the routine use group. We evaluated the time to CT in all groups and considered the reasons for the delays in the transition and routine use groups.

**Results:**

On sliding gantry CT implementation, although time to CT remained unaffected in the transition group, it significantly reduced in the routine use group, independent of injury severity score. The incidence of cable management problems was significantly higher in the latter group.

**Conclusions:**

We have demonstrated a decrease in the time to CT with the implementation of a sliding gantry CT. However, due to a higher number of cable management problems in the routine use group, we recommend regular refresher team training with routine use.

## Background

Severe injury is one of the most common causes of death worldwide in patients aged between 20 and 40 years [1, 2]. Furthermore, hemorrhage is responsible for a large number of deaths during the prehospital and early hospital phases [3]. Therefore, rapid detection of bleeding and its source is the most important diagnostic aspect to reduce mortality in severely injured patients [4, 5].

In trauma suite diagnostics and treatment, following the “Advanced Trauma Life Support” algorithm, bleeding in the four compartments (thorax, abdomen, pelvis, and femoral) and external bleeding is detected by clinical examination supported by the “Focused Assessment with Sonography in Trauma (FAST)” as an adjunct in the primary survey [6]. FAST has a steep learning curve, and it can detect free fluid with a sensitivity of 50–60% and specificity of 90–100% [7–9]. Walcher et al. reported that within 1 day of hands-on training, participants were able to perform ultrasound procedures at the scene of an accident with a high level of accuracy [9].

The most important diagnostic tool to detect bleeding and its source during the secondary survey is whole-body CT (WBCT) [6, 10, 11]. Implementation of multi-slice CT in the treatment algorithm could significantly reduce the time in the trauma suite [5]. In comparison to conventional radiography, CT enables a faster and more consistent diagnosis [12]. However, implementing multi-slice CT in the trauma suite algorithm has some crucial prerequisites. In addition to the organizational problems of scanning the patient in radiology, the CT suite must be equipped for hemodynamic resuscitation of injured patients [12]. To address these problems, the number of trauma suites equipped with CT has increased in the last few years [13]. Furthermore, trauma suite CT (tr-CT) could decrease time in the trauma suite, which is crucial for reducing mortality [13–15]. Now, dual-room twin-CT scanners with a sliding gantry CT in the middle of two carbon CT tables are available for trauma suites [16]. In particular, a sliding gantry CT in the trauma suite significantly reduces the time to CT with the same throughput as two separate CT units [14].

However, there are several obstacles to implementing tr-CT. In this study, we retrospectively investigated the time to CT and the potential obstacles to using the sliding gantry tr-CT, assuming that the former would be reduced after training the trauma suite staff to use the new algorithm and infrastructure.

## Materials and methods

### Study design

We included all trauma suite treatments using multi-slice CT from January 2016 to December 2017 in the study. Time from admission in the trauma suite to first CT imaging, the injury severity score (ISS), and events leading to a delay in the trauma suite treatment were analyzed anonymously. The events were captured for internal quality improvement and then analyzed retrospectively by grouping for cable management issues, patient positioning, table issues, staff issues, and hardware/software problems. For trauma suite treatment, the “Advanced Trauma Life Support” algorithm was used. The methodology used in this study adheres to the Strengthening the Reporting of Observational Studies in Epidemiology (STROBE) guidelines.

### Implementation of the tr-CT and grouping

Trauma suite treatments from January to December 2016 formed the control group. We used a conventional CT placed in the Department of Radiology, 44.6 m from the trauma suite. During this period, only the time to CT and ISS were analyzed.

Both trauma suites were modified from January to April 2017, and a sliding gantry CT (SOMATOM Definition AS+ , Siemens Healthcare AG, Erlangen, Germany) was installed in the suites (interim group). During this period, the interim trauma suite, located 54.8 m away from the conventional CT scanner, was used. Here, only the time to CT and ISS were analyzed.

**Fig. 1:**
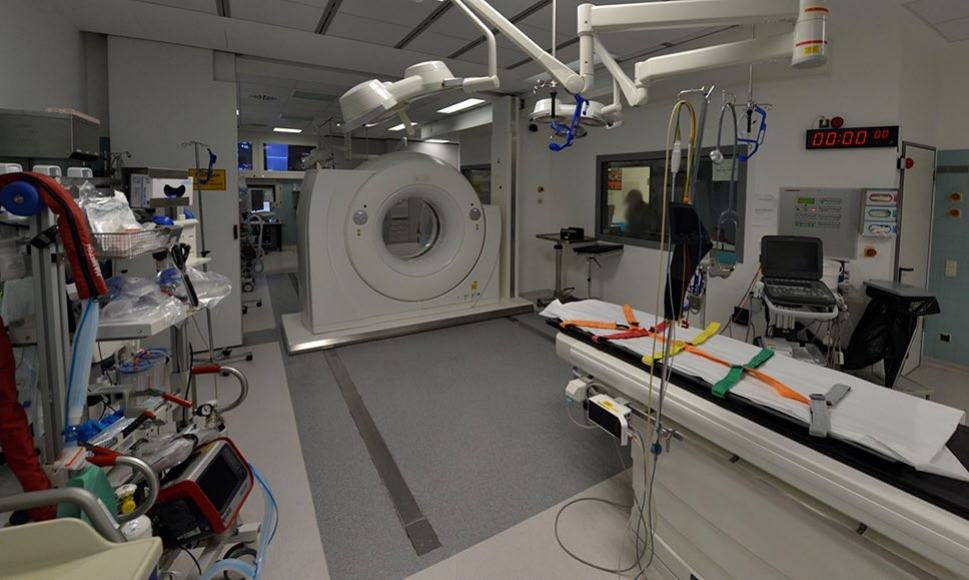
Overview of the trauma suite. The two trauma suites are equipped with a dual-room sliding gantry CT scanner in the middle of the two trauma suites. The rooms are separated by a radiation protection door. Both trauma suites are equipped equally

Trauma suites with attached sliding gantry CT have been used since May 2017 (Fig. 1). The first three months (May to July 2017) were considered the transition period (transition group). Treatments from August to December 2017 formed the routine use group. The time to CT, events leading to a delay, and ISS were analyzed in the transition and routine use groups.

We sub-grouped the patients based on ISS to reduce the bias related to injury severity. Patients with an ISS of ≥ 16 were assumed to be severely injured [17, 18], while others were considered slightly injured.

### Statistical analyses

All data are presented as mean ± standard deviation (SD) for normally distributed variables and as median with interquartile range (IQR) for non-normally distributed variables. Normal distributions were verified using the Kolmogorov–Smirnov test. We used SPSS Statistics version 26 (IBM, Armonk, NY, USA). The Kruskal–Wallis test was used for groupwise comparison and the Fisher's test for categorical data. Statistical significance was set at *p* < 0.05.

### Ethics approval and consent to participate

This study involved a retrospective analysis of anonymized data collected during regular emergency department (ED) treatment. As per the general terms and conditions of the treatment contract of the University Hospital of Magdeburg, this study did not require ethical approval.

## Results

We evaluated 436 trauma suite treatments, comprising 309 patients with ISS < 16 and 127 patients with ISS ≥ 16. The patients included 127 women (mean age = 45.5 ± 22.9 years) and 309 men (mean age = 45.0 ± 18.8 years). The control, interim, transition, and routine use groups included 141, 56, 77, and 162 patients, respectively. Table 1 summarizes the detailed patient and subgroup characteristics.

**Table 1:** Patients and subgroup characteristics

	Males	Mean age (years)	Females	Mean age (years)
Overall				
ISS < 16	216	42.8 ± 18.2	89	42.1 ± 21.1
ISS ≥ 16	93	50.3 ± 19.1	38	53.6 ± 25.4
Overall	309	45.0 ± 18.8	127	45.5 ± 22.9
Control group				
ISS < 16	59	42.7 ± 18.2	20	38.1 ± 20.4
ISS ≥ 16	45	43.8 ± 16.9	17	46.1 ± 24.3
Overall	104	43.1 ± 17.6	37	41.7 ± 22.3
Interim group				
ISS < 16	29	42.3 ± 18.3	19	44.8 ± 15.5
ISS ≥ 16	6	57.0 ± 15.3	2	40.5 ± 46.0
Overall	35	44.8 ± 18.5	21	44.4 ± 18.0
Transition group				
ISS < 16	43	42.2 ± 19.0	12	50.8 ± 22.1
ISS ≥ 16	16	51.4 ± 19.6	6	59.3 ± 20.5
Overall	59	45.5 ± 19.3	18	53.7 ± 21.4
Routine use group				
ISS < 16	85	42.8 ± 18.1	38	40.1 ± 23.2
ISS ≥ 16	26	59.4 ± 19.5	13	62.7 ± 25.1
Overall	111	46.7 ± 19.7	51	45.8 ± 25.4

### Time from admission to sliding gantry CT

**Fig. 2:**
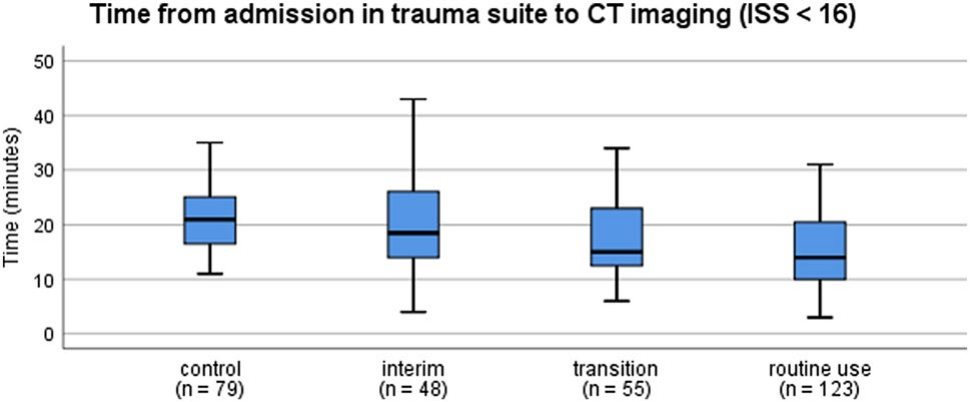
Time from admission in trauma suite to CT imaging (ISS < 16). The box plots indicate the time from admission to CT imaging in patients with ISS < 16. Compared to the control group, the routine use group shows a significant decrease in time to CT (*p* < 0.001; Kruskal–Wallis test). Box: interquartile range; whiskers: minimum and maximum values

**Fig. 3:**
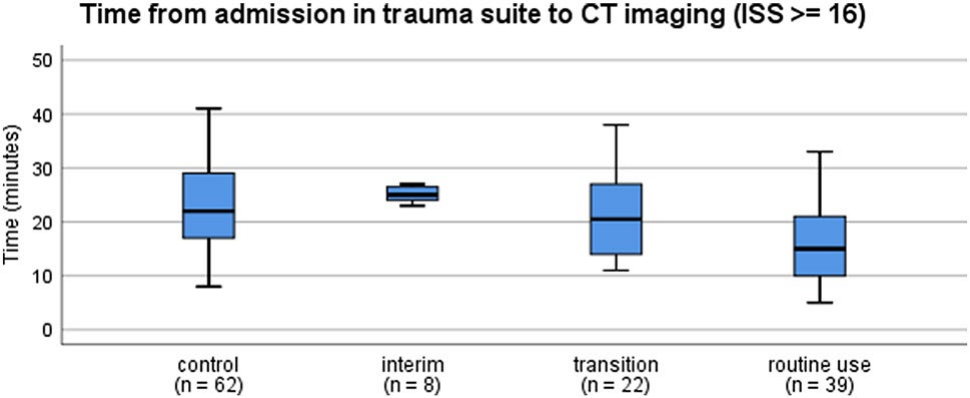
Time from admission in trauma suite to CT imaging (ISS ≥ 16). The box plots indicate the time from admission to CT imaging in patients with ISS ≥ 16. Compared to the control group, the routine use group shows a significant decrease in time to CT (*p* = 0.002; Kruskal–Wallis test). Box: interquartile range; whiskers: minimum and maximum values

No significant difference was seen in the time from admission to CT between the control and interim groups [ISS < 16, 21.0 min (IQR 9 min) vs. 18.5 min (IQR 13 min) *p* = 1.0, and ISS ≥ 16, 22 min (IQR 12 min) vs. 25 min (IQR 3 min), *p* = 1.0]. In the transition group, the time to CT was decreased [ISS < 16: 15 min (IQR 12 min); ISS ≥ 16: 20.5 min (IQR 14 min)] though the difference was not significant. Compared to the control group, the routine use group with the sliding gantry CT showed a significant decrease in time to CT [ISS < 16: 14.0 min (IQR 11 min), *p* < 0.001, Fig. 2, and ISS ≥ 16: 15.0 min (IQR 12 min), *p* = 0.002, Fig. 3].

### Reasons for delay in trauma suite treatment in the sliding gantry CT group

**Fig. 4:**
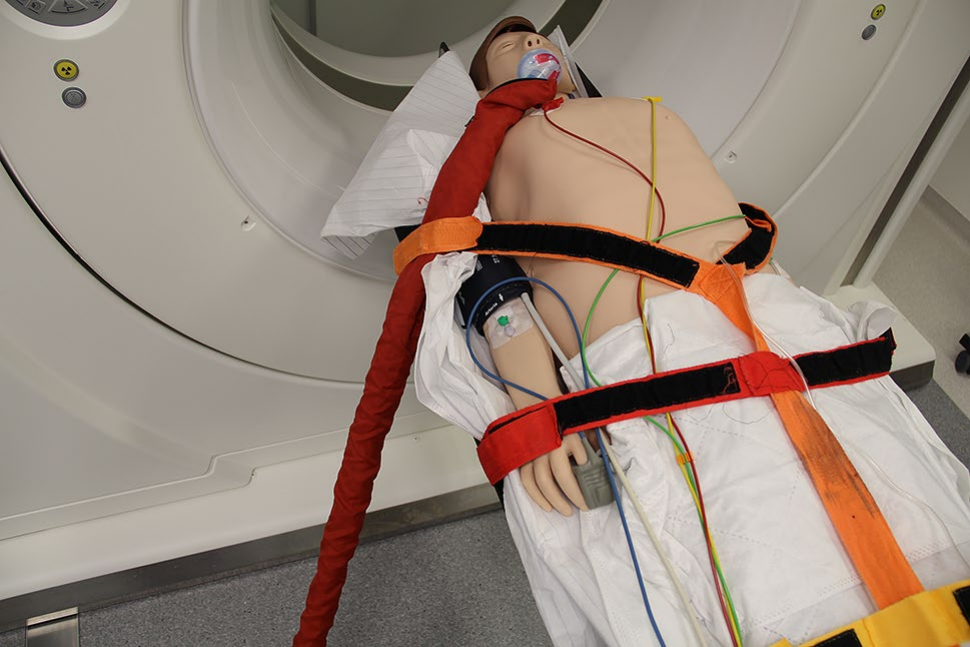
Correct cable management demonstrated on a patient simulator. The correct cable management is crucial for a fast CT scan. We found an increase in cable management issues in the routine use group causing delay to CT scan (e.g., by causing collision alarm and abort of the CT scan)

We analyzed the reasons for delay in trauma suite treatments in a blinded manner in the transition and routine use groups (Table 2). A significant increase in cable management issues (delaying positioning of monitoring cable during preparation of the table for CT scan or abort of the CT scan due to irritating cables, Fig. 4) was observed in the routine use group when compared to the transition group (14.2% vs. 3.9%; *p* = 0.015). However, there were no significant differences in patient positioning (3.9% vs. 8.0%; *p* = 0.281), table issues (5.2% vs. 5.6%; *p* = 1.0), staff issues (1.3% vs. 1.9%; *p* = 1.0), or hardware/software problems (0.0% vs. 3.7%; *p* = 0.181).

**Table 2:** Issues that cause a delay in trauma suite treatment

	Transition group (*n* = 77) (%)	Routine use group (*n* = 162) (%)
Cable management	3.9	14.2
Patient positioning	3.9	8.0
Table issues	5.2	5.6
Staff issues	1.3	1.9
Hardware/software problems	0.0	3.7

## Discussion

We evaluated the influence of implementing a sliding gantry CT in the trauma suite on the diagnostic interval and found a significant decrease in the time taken from admission to CT in the routine use group, after a period of implementation.

WBCT is an important part of trauma suite diagnostics, as it contributes to reducing mortality in severe blunt trauma cases [11, 19, 20]. The time to CT is a well-evaluated quality indicator for trauma suite treatment [21]. Implementation of multi-slice CT in the treatment algorithm can shorten the time in the trauma suite [5]. Further, implementing a tr-CT scanner reduces the time in the trauma suite, which could be crucial for reducing mortality [13–15]. In particular, a sliding gantry CT in the trauma suite has been reported to significantly reduce the time to CT with the same throughput as two separate CT units [14], which is consistent with our findings. However, the sliding gantry CT implementation effect was evident after a short period of three months. In this study, we identified problems that could arise during the implementation. Although the routine use group showed a significant increase in cable management issues compared to the transition group, the time to CT was less in the routine use group. This could be because the end-user training took place just before the start of the transition period. The focus of this training was cable management strategy, table usage, and initial team positioning. Every physician and nurse working in the trauma suite had to undergo this initial training. Therefore, small teams were built randomly, and a simulation patient was transferred to the trauma suite table, where the primary survey of the ATLS was conducted. The training was finalized before CT scan, including the preparation of the trauma suite table for CT scan. Based on our findings, we strongly recommend a refresher training three months after implementing the tr-CT. The refresher training should include the same contents, addressing especially the new members of the trauma suite team. Although the results were not statistically significant, there was a trend towards more software/hardware issues in the routine use group. Thus, it seems reasonable to actively involve the CT supplier in the clinical implementation process and refresher training.

Despite the problems, the implementation of tr-CT significantly reduced the time to CT during its routine use. This is consistent with the study by Huber-Wagner et al. showing the benefits of WBCT in severely injured patients [11, 20]; additionally, reducing the time to WBCT reduces the mortality in blunt trauma cases [19]. Furthermore, Furugori et al. have shown that the implementation of a tr-CT could further reduce the time to WBCT [13]. Consistent with these previous reports, we also noted a reduction in time to WBCT after a transition period of three months. Kippnich et al. found an increase in time to WBCT after implementation of a dual-room twin-CT-scanner trauma suite [16]. Previously, the CT was in a single-room trauma suite [16]. They explained this fact by lack of sufficient training for the trauma suite team [16]. As we executed team trainings prior to usage of the tr-CT, the time to WBCT was similar for both the transition and control groups. Moreover, the time to WBCT significantly decreased in the routine use group.

Our study has some limitations. First, we analyzed only structured and anonymized data on the causes for delay. Therefore, detailed descriptions of the problems were not addressed in this study. However, they were communicated immediately following their occurrence in the related departments (e.g., trauma surgery, radiology, anesthesiology, neurosurgery, and visceral surgery). Last, this study only investigated the surrogate parameter “time from admission to WBCT.”

## Conclusions

Several challenges were associated with the implementation of the sliding gantry tr-CT scanner. Here, we used team training to avoid the common issues. However, the time to CT in the transition group was the same before and immediately after implementation of the tr-CT scanner. The effects were first seen during the routine use period, which in this study was three months after implementation. However, the routine use group had still significant problems in cable management. Therefore, regular refresher training seems reasonable. Addressing these implementation problems reduced the time to WBCT significantly after a short transition period of three months. The influence of reducing time to WBCT on clinical outcomes should be further addressed in a randomized trial.

## Data Availability

The datasets generated and/or analyzed during the current study are not publicly available due to data privacy rules but are available from the corresponding author upon reasonable request.
